# Recruitable alveolar collapse and overdistension during laparoscopic gynecological surgery and mechanical ventilation: a prospective clinical study

**DOI:** 10.1186/s12871-022-01790-7

**Published:** 2022-08-06

**Authors:** Mantas Dargvainis, Henning Ohnesorge, Dirk Schädler, Ibrahim Alkatout, Inéz Frerichs, Tobias Becher

**Affiliations:** 1 Department of Anesthesiology and Intensive Care Medicine, University Medical Center Schleswig-Holstein, Campus Kiel, Kiel, Germany; 2 Department of Gynecology and Obstetrics, University Medical Center Schleswig-Holstein, Campus Kiel, Kiel, Germany

**Keywords:** Electrical Impedance Tomography, Lung-Protective Ventilation, Positive End-Expiratory Pressure, Postoperative Pulmonary Complications, Laparoscopic Surgery, Trendelenburg position

## Abstract

**Background:**

Laparoscopic surgery in Trendelenburg position may impede mechanical ventilation (MV) due to positioning and high intra-abdominal pressure. We sought to identify the positive end-expiratory pressure (PEEP) levels necessary to counteract atelectasis formation (“Open-Lung-PEEP”) and to provide an equal balance between overdistension and alveolar collapse (“Best-Compromise-PEEP”).

**Methods:**

In 30 patients undergoing laparoscopic gynecological surgery, relative overdistension and alveolar collapse were assessed with electrical impedance tomography (EIT) during a decremental PEEP trial ranging from 20 to 4 cmH_2_O in supine position without capnoperitoneum and in Trendelenburg position with capnoperitoneum.

**Results:**

In supine position, the median Open-Lung-PEEP was 12 (8–14) cmH_2_O with 8.7 (4.7–15.5)% of overdistension and 1.7 (0.4–2.2)% of collapse. Best-Compromise-PEEP was 8 (6.5–10) cmH_2_O with 4.2 (2.4–7.2)% of overdistension and 5.1 (3.9–6.5)% of collapse. In Trendelenburg position with capnoperitoneum, Open-Lung-PEEP was 18 (18–20) cmH _2_ O (*p* < 0.0001 vs supine position) with 1.8 (0.5–3.9)% of overdistension and 0 (0–1.2)% of collapse and Best-Compromise-PEEP was 18 (16–20) cmH_2_O (*p* < 0.0001 vs supine position) with 1.5 (0.7–3.0)% of overdistension and 0.2 (0–2.7)% of collapse. Open-Lung-PEEP and Best-Compromise-PEEP were positively correlated with body mass index during MV in supine position but not in Trendelenburg position.

**Conclusion:**

The PEEP levels required for preventing alveolar collapse and for balancing collapse and overdistension in Trendelenburg position with capnoperitoneum were significantly higher than those required for achieving the same goals in supine position without capnoperitoneum. Even with high PEEP levels, alveolar overdistension was negligible during MV in Trendelenburg position with capnoperitoneum.

**Trial registration:**

This study was prospectively registered at German Clinical Trials registry (DRKS00016974).

## Background

Postoperative pulmonary complications (PPC) are common after general anesthesia with mechanical ventilation and may lead to prolonged hospitalisation, increased mortality and morbidity [1]. Increased pressure (barotrauma) or high volumes (volutrauma) during mechanical ventilation may damage lung parenchyma by causing high transpulmonary pressures that exceed the elastic capacity of the lungs. Further damage to lung parenchyma may be caused by cyclic opening and closing of alveolar units during inspiration and expiration (atelectrauma) [2, 3]. Therefore, adequate lung protective ventilation should be applied in order to reduce the risk of PPC [4, 5]. While low tidal volume became an established component of lung protective ventilation, the optimal PEEP value for preventing alveolar collapse and regional hypoventilation without causing overdistension during general anesthesia remains controversial [6, 7]. Additionally, possible hemodynamic effects of higher PEEP levels requiring more intravenous fluids and vasoactive medications should be taken into consideration while choosing the most appropriate PEEP value for lung protective ventilation [6]. The situation is even more complicated in case of laparoscopic surgery in Trendelenburg position due to high pleural pressure caused by positioning and capnoperitoneum [8] and may require higher PEEP values for preventing alveolar collapse in comparison to ventilation in supine position without capnoperitoneum [9].

Unfortunately, standard monitoring parameters used during mechanical ventilation, such as peripheral oxygen saturation (SpO_2_), are not suitable for identifying regional phenoma such as atelectasis formation and pulmonary overdistension during mechanical ventilation. Electrical impendence tomography (EIT) is a noninvasive and radiation-free imaging modality based on the measurement of electrical potentials at the chest wall surface that can be used for direct monitoring of regional ventilation [10–12]. Analysing tidal impedance changes during MV with decremental PEEP titration allows assessment of regional alveolar collapse and overdistension. This information can be used for identifying individualized PEEP levels that minimize both overdistension and atelectasis formation [13]. In a randomized trial, Pereira and colleagues showed that ventilation with EIT-guided individualized PEEP resulted in a reduction of postoperative atelectasis in comparison to ventilation with a fixed PEEP of 4 cmH_2_O [14]. In that study, PEEP trials were performed for EIT-guided identification of PEEP levels in a subgroup of ten patients undergoing laparoscopic surgery. However, PEEP trials were only conducted after the induction of general anesthesia and not after the establishment of capnoperitoneum. Ukere and colleagues demonstrated that hypoventilated areas detected using EIT were increased by MV, capnoperitoneum and Trendelenburg positioning in patients undergoing laparoscopic surgery [15]. Both aforementioned studies, did not seek to identify the actual PEEP levels necessary to counteract atelectasis formation and to minimize overdistension during Trendelenburg position with capnoperitoneum.

We hypothesized that the PEEP levels required to achieve these goals would be significantly higher in Trendelenburg position with capnoperitoneum as compared to supine position after induction of general anesthesia. Thus, we conceived a prospective clinical study using EIT to detect the lowest PEEP level without signs of alveolar collapse (“Open Lung PEEP”) as well as the PEEP level associated with equal amounts of overdistension and alveolar collapse (“Best Compromise PEEP”) in Trendelenburg position with capnoperitoneum and compare these results with the PEEP values needed to reach the same conditions in supine position without capnoperitoneum. Additionally, we assessed the hemodynamic changes associated with the abovementioned PEEP levels in comparison to baseline taken after induction of general anesthesia.

## Methods

We conducted a prospective clinical study in the Department of Gynecology and Obstetrics at the University Medical Center Schleswig–Holstein, Campus Kiel, Germany. The study received approval from the Ethics Committee of the Medical Faculty of Christian-Albrechts-University Kiel, Germany (D426/19) and was registered at German Clinical Trials registry on 14/03/2019 before including the first patient (DRKS00016974). 30 patients were enrolled between March 2019 and January 2020. Written informed consent was obtained from all study participants. We included patients aged more than 18 years with scheduled laparoscopic surgery in Trendelenburg position under general anesthesia. Patients with body mass index (BMI) above 35 kg/m^2^, presence of metallic implants or open injuries in the thoracic area, suspected or confirmed pregnancy, chronic pulmonary diseases or functional capacity below four metabolic equivalents of activity were excluded.

### Anesthesiologic management

General anesthesia was induced with Sufentanil (0.2–0.4 µg/kg), Propofol (1.5–2 mg/kg) and Rocuronium (0.6 mg/kg) and maintained with Sufentanil and Sevoflurane according to routine clinical practice in our institution. Fluid replacement was conducted with balanced crystalloid solution according to the treating anesthesiologist’s discretion (usually 0.5 l crystalloid solution per hour of surgery). Patients were ventilated in a pressure-controlled mode with inspiratory pressure adjusted to achieve a tidal volume of 6–8 ml per kg ideal body weight and respiratory rate of 12–15/min, applying a PEEP of 5 cmH_2_O in supine position and 8 cmH_2_O in Trendelenburg position with capnoperitoneum.

### Study procedure

Before induction of general anesthesia, a 16-electrode belt was attached to the patient’s chest circumference at the level of the 4^th−^6^th^ intercostal space, measured at the parasternal line. EIT measurements were conducted at a scan rate of 50 images per second using the PulmoVista 500 EIT device (Dräger, Lübeck, Germany). Patients were intubated with an endotracheal tube of 7.5 mm internal diameter. EIT data were recorded under two conditions: 1) supine position without capnoperitoneum after induction of general anesthesia; 2) 30° Trendelenburg position after establishment of capnoperitoneum with a positive intra-abdominal pressure of 10–12 mmHg. In each of these two conditions, a decremental PEEP trial was conducted measuring alveolar collapse and overdistension. For this purpose, the PEEP level was first increased to 20 cmH_2_O in two or three one-minute steps. Subsequently, the set PEEP was decreased in one-minute steps of 2 cmH_2_O from 20 cmH_2_O to 4 cmH_2_O. During the collection of EIT data, vital parameters such as heart rate, arterial pressure and SpO_2_ were registered at baseline (directly after induction of general anesthesia in supine position without capnoperitoneum while ventilating the patients with PEEP of 5 cmH_2_O) and during each step of the PEEP trial. Additionally, the use of vasoactive medications was noted. After the end of the PEEP trial, mechanical ventilation was continued with clinically selected PEEP levels (usually 5 cmH_2_O during supine position and 8 cmH_2_O during Trendelenburg position with capnoperitoneum).

### Analysis of EIT data

EIT data was analysed offline using the EIT Data Analysis Tool 6.1 and PulmoVista PC Software 1.2 (both Dräger, Lübeck, Germany), and Microsoft Excel 2010 (Microsoft, Seattle, WA, USA). The percentages of alveolar collapse and overdistension for each PEEP level were calculated using the algorithm proposed by Costa and colleagues [13] as implemented in the PulmoVista Software. Respiratory system compliance (C_rs_) was calculated by dividing tidal volume by the corresponding driving pressure. For calculation of pixel C_rs_, local tidal volume was set equal to local tidal impedance change and driving pressure was estimated in pressure-controlled mode by subtracting PEEP from the set inspiratory pressure.

Open Lung PEEP was defined as the lowest PEEP level with less than or equal to 3% of alveolar collapse. Best Compromise PEEP was defined as the PEEP level associated with equal amounts of alveolar overdistension and alveolar collapse.

### Study objectives

The primary objective of the study was to identify the lowest PEEP level at which no alveolar collapse could be detected with EIT (“Open Lung PEEP”) in a 30° Trendelenburg position with capnoperitoneum. Further objectives were to identify the PEEP level with equal amounts of overdistension and alveolar collapse (“Best compromise PEEP”) in the 30° Trendelenburg position with existing capnoperitoneum, to identify Open Lung PEEP and Best Compromise PEEP in the horizontal supine position and to describe the PEEP level with the highest global respiratory system compliance. Furthermore, we sought to assess blood pressure, heart rate and oxygen saturation at the abovementioned PEEP levels and to investigate the correlation between Open Lung PEEP, Best Compromise PEEP and BMI.

### Statistical analysis

To obtain solid estimates for Open Lung PEEP and Best Compromise PEEP, adequately reflecting the clinical spectrum of weight characteristics in the range below BMI 35, we estimated that a sample size of 30 patients would be suitable. Statistical analysis was performed using GraphPad Prism 8.0 (GraphPad Software, La Jolla, CA, USA). All data sets were tested for normal distribution using D’Agostino Pearson omnibus normality test. Normally distributed variables are reported as mean with standard deviation, and not normally distributed variables as median with 25-75th percentile. Comparisons were performed using the paired t-test for normally distributed data and Wilcoxon matched pairs test for not normally distributed data. For comparison of repeated measurements, Friedman’s test with Dunn’s post hoc analysis was used. For comparison of categorical variables, Fisher exact test was applied.

## Results

**Fig. 1:**
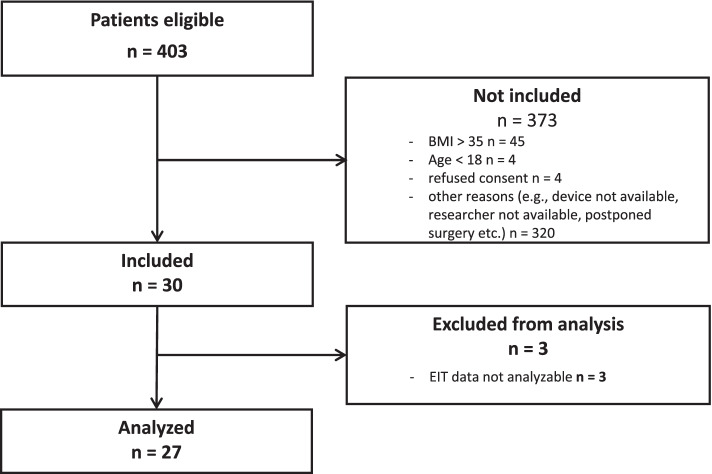
CONSORT diagram. BMI = Body Mass Index; EIT = Electrical Impedance Tomography

Twenty-seven patients with completed sets of EIT measurements were included in the final analysis. Patient flow is presented in the CONSORT diagram (Fig. 1). 3 of initially enrolled 30 patients had to be excluded from the analysis due to poor quality of EIT measurements caused by use of electrocautery during the PEEP trial in Trendelenburg position with capnoperitoneum. All study participants were females with mean age of 35.8 ± 12.6 years. Baseline clinical characteristics of analysed patients are summarised in Table 1.

**Table 1:** Baseline clinical characteristics of patients with complete sets of Electrical Impedance Tomography measurements (*N* = 27)

Parameters	Results
Age (years)	35.8 ± 12.6
Weight (kg)	74.5 ± 16.4
Height (cm)	168 ± 8
BMI (kg/m^2^)	26.5 ± 5.5
Main diagnosis (%)	
- Endometriosis	27
- Myomas	20
- Cystic changes	17
- Adhesions	13
- Other	23
Operation duration (min)	74 (70–82)

*BMI* Body mass index

**Fig. 2:**
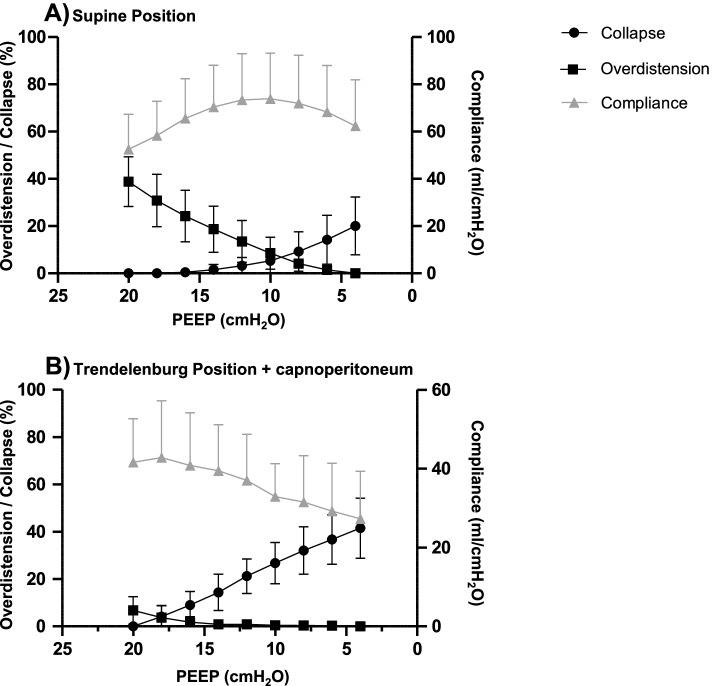
Changes of alveolar collapse, overdistension and global respiratory system compliance during a PEEP trial (beginning from 20 cmH_2_O to 4 cmH_2_O in steps of 2 cmH_2_O every minute) in **A**) supine position without capnoperitoneum and **B**) Trendelenburg position with capnoperitoneum. Each point represents mean value of all 27 patients and whiskers show the standard deviation. Overdistension decreases towards lower PEEP values, while alveolar collapse increases simultaneously. The intersection between the lines representing alveolar collapse and overdistension indicates Best Compromise PEEP

**Fig. 3:**
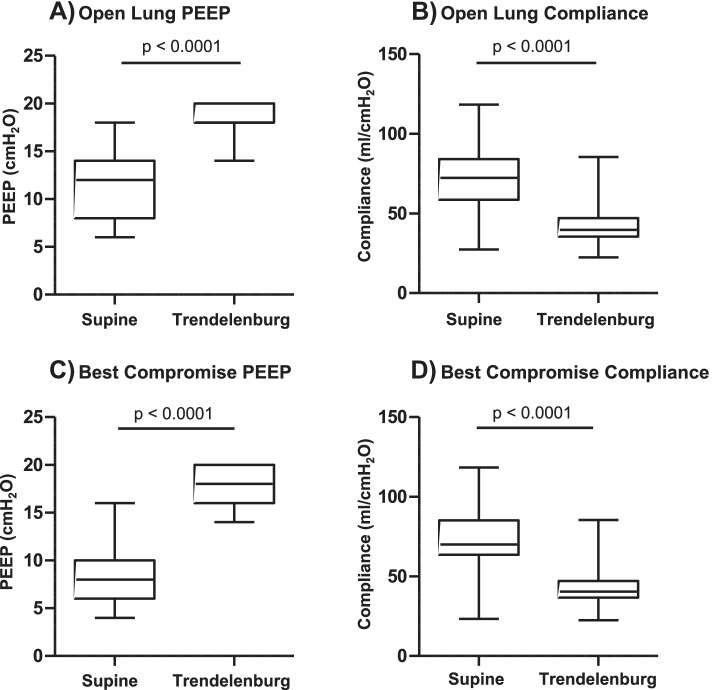
Comparison of PEEP and respiratory system compliance between Trendelenburg position with capnoperitoneum and supine position without capnoperitoneum under ventilation with Open Lung PEEP (**A**-**B**) and Best Compromise PEEP (**C**-**D**). Box plot (median with 25 and 75 percentiles) with whiskers showing range

In supine position without capnoperitoneum, Open Lung PEEP was established at 12 (8–14) cmH_2_O. In Trendelenburg position with capnoperitoneum, the same criterion was fulfilled under ventilation with a PEEP of 18 (18–20) cmH_2_O (*p* < 0.0001 vs supine position). Best Compromise PEEP was also reached with significantly higher PEEP values of 18 (16–20) cmH_2_O in Trendelenburg position with capnoperitoneum in comparison to 8 (6.5–10) cmH_2_O in supine position without capnoperitoneum (*p* < 0.0001). Figure 2 depicts the decremental PEEP trial in supine and Trendelenburg positions. Global C_rs_ was significantly lower in Trendelenburg position with capnoperitoneum in comparison to supine position without capnoperitoneum (*p* < 0.0001) for both Open Lung and Best Compromise PEEP (Fig. 3), while tidal volume showed no relevant difference between positioning.

In supine position without capnoperitoneum, Open Lung PEEP was significantly higher than Best Compromise PEEP (*p* < 0.0001), while there was no significant difference between Open Lung PEEP and Best Compromise PEEP in Trendelenburg position with capnoperitoneum (*p* = 0.09).

**Fig. 4:**
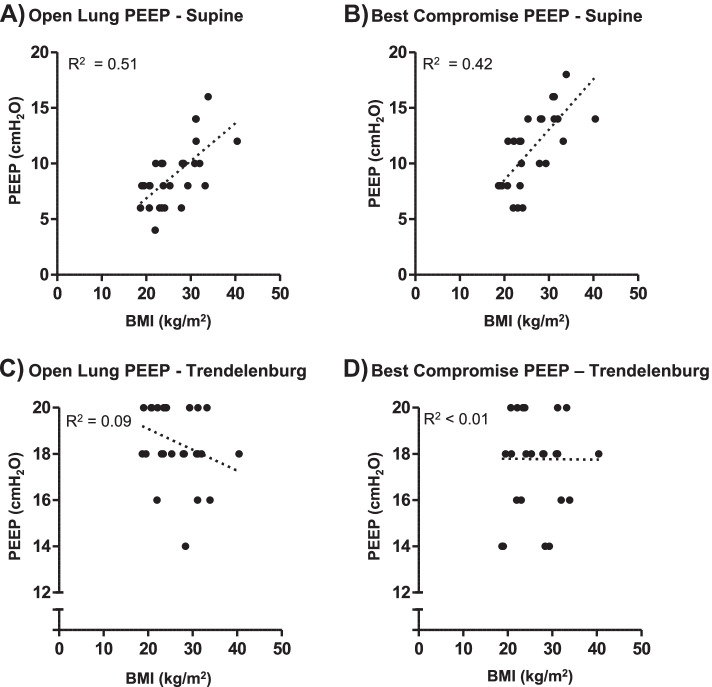
Correlation between body mass index (BMI) and PEEP in supine position without capnoperitoneum (**A**, **B**) and Trendelenburg position with capnoperitoneum (**C**, **D**). Dotted lines represents the best fit. Open Lung PEEP (Supine) = 0.45 * BMI—0.6 (*R*^2^ = 0.51; *p* < 0.0001). Best Compromise PEEP (Supine) = 0.34 * BMI + 0.1 (*R*^2^ = 0.42; *p* = 0.0003). The correlation between PEEP and BMI was not statistically significant in Trendelenburg position

Both Open Lung PEEP and Best Compromise PEEP were positively correlated with BMI during mechanical ventilation in supine position (*r*^2^ = 0.51, *p* < 0.0001 for Open Lung PEEP and *r*^2^ = 0.42, *p* = 0.0003 for Best Compromise PEEP) but not in Trendelenburg position with capnoperitoneum (Open Lung PEEP *p* = 0.09 and Best Compromise PEEP *p* = 0.8; Fig. 4). According to our data, Best Compromise PEEP in supine position could be calculated using the linear regression equation: PEEP = 0.34 * BMI + 0.1 (Fig. 4) and Open-Lung PEEP could be calculated according to the equation PEEP = 0.45 * BMI – 0.6.

In the Trendelenburg position with capnoperitoneum, a lower heart rate of 60 (54–65) beats/min and higher mean arterial blood pressure of 89.5 (83–98.3) mmHg were documented with Open Lung PEEP in comparison to baseline (heart rate 68 (62–76) beats/min, mean arterial blood pressure 71.5 (64.8–81.8 mmHg) and supine position (heart rate 66 (59–75) beats/min, mean arterial blood pressure 73.0 (69.3–81.3) mmHg) (*p* < 0.0001). Similar results were also observed while ventilating with Best Compromise PEEP with lower heart rate and higher mean arterial blood pressure in Trendelenburg position compared to baseline and supine position (*p* < 0.0001). There was no significant difference in SpO_2_ between different positions and baseline. During PEEP trial, 3 of 27 patients needed vasoactive drugs in Trendelenburg position with capnoperitoneum and 14 of 27 patients in supine position (*p* = 0.0001).

The average SpO_2_ in the recovery room was 95.5 ± 2.3% without supplemental oxygen. None of the patients investigated in this study had additional oxygen requirements or needed invasive or non-invasive mechanical ventilation after the end of surgery.

## Discussion

In our study, we found that both the PEEP level required for preventing alveolar collapse (“Open Lung PEEP”) as well as the PEEP level required for equally balancing overdistension and collapse (“Best Compromise PEEP”) was significantly higher in Trendelenburg position with capnoperitoneum than in supine position. Both Open Lung PEEP and Best Compromise PEEP were positively correlated with BMI in supine position but not in Trendelenburg position with capnoperitoneum.

Currently, there is no international consensus on best PEEP level for prevention of regional hypoventilation and PPC. Frequently, a conservative PEEP level of less than 5 cmH_2_O is being used for ventilation both in open abdominal and in laparoscopic surgery [16]. Our findings imply that to minimize alveolar collapse and overdistension during MV under general anesthesia, higher PEEP values in the range of 16 to 20 cmH_2_O can be considered in Trendelenburg position with capnoperitoneum, without additional risk for hemodynamic compromise or lung overdistension. BMI could be used as additional criterium for choosing individualised PEEP value in supine position without capnoperitoneum using the regression equations presented in Fig. 4. For Best Compromise PEEP, the regression equation can be simplified with reasonable accuracy as follows: Best Compromise PEEP = BMI / 3, yielding an equation that is easily applicable at the bedside.

Our seemingly contradictory finding of a positive correlation between the required PEEP value and BMI in the supine position but not in the Trendelenburg position with capnoperitoneum requires cautious interpretation. The mechanism by which a higher BMI leads to higher PEEP requirements is most likely closely related to intra-abdominal pressure, which is positively correlated to BMI during general anesthesia [17]. In our study, abdominal pressure during capnoperitoneum was closely monitored and kept at a steady level of 10–12 mmHg throughout the course of surgery whenever possible. This may have at least partially offset differences in intra-abdominal pressure due to BMI. On the other hand, we cannot entirely exclude the possibility that in Trendelenburg position with capnoperitoneum, some patients would have required even higher PEEP levels than the ones investigated in our study (20 – 4 cmH_2_O). Due to the nature of the EIT analysis performed for the present study, “Best Compromise PEEP” and “Open Lung PEEP” in these patients would still be identified within the range of 20 – 4 cmH_2_O, precluding any possible differentiation in the higher PEEP range.

Our suggestion of using higher PEEP values for MV in Trendelenburg position is in line with the findings of another study on EIT-guided PEEP ventilation conducted by Pereira and colleagues [15]. They describe PEEP values of 13.5 ± 1.6 cmH_2_O for counteraction of atelectasis formation in Trendelenburg position, although the PEEP trial was conducted before establishment of capnoperitoneum. This may explain the higher PEEP values for prevention of alveolar collapse and equally balancing overdistension and collapse reported in our study. Nevertheless, Pereira and colleagues were able to demonstrate the positive effect of EIT guided ventilation using postoperative CT scans showing less atelectasis formation compared to a control group ventilated with a fixed PEEP of 4 cmH_2_O.

Investigating patients undergoing robot-assisted laparoscopic prostatectomy with capnoperitoneum in Trendelenburg position, Shono and colleagues reported that a higher PEEP level of 15 cmH_2_O had a positive effect on regional ventilation distribution as compared to ventilation with PEEP of 5 cmH_2_O [18]. However, despite more homogeneous intraoperative ventilation no relevant improvement of postoperative lung function could be observed while comparing two patient groups ventilated with PEEP of 15 and 5 cmH_2_O.

Another study on optimal PEEP level in Trendelenburg position with capnoperitoneum during laparoscopic surgery used ventilation parameters such as dynamic compliance, dead space to tidal volume ratio and intrapulmonary shunt ratio for evaluation of lung-protective ventilation and suggested ventilation with a moderate PEEP of 8 cmH_2_O for improvement of pulmonary ventilation [9]. In this study, a lower PEEP level of 0 or 4 cmH_2_O was argued to be not effective to compensate the effects of capnoperitoneum and Trendelenburg position, while a higher PEEP of 12 cmH_2_O was associated with increased intrapulmonary shunt and hemodynamic depression. In contrast, we observed preserved oxygenation and hemodynamic stability with the application of EIT-guided PEEP in Trendelenburg position with capnoperitoneum. All in all, high PEEP values during ventilation with Trendelenburg position with capnoperitoneum were well tolerated in our study.

Karsten and colleagues suggested ventilation with PEEP of 10 cmH_2_O for prevention of regional hypoventilation in patients undergoing laparascopic surgery in supine position while monitoring regional ventilation with EIT [19]. These results corresponded to our Open Lung and Best Compromise PEEP values established in supine position without capnoperitoneum.

Erlandsson and co-workers suggested ventilation with PEEP 15 ± 1 cmH_2_O during laparoscopic surgery in supine position for morbidly obese patients with BMI 49 ± 8 kg/m^2^ according to monitoring of regional ventilation with EIT, which was performed before the beginning of surgery and induction of capnoperitoneum [20]. These values are higher than the ones observed in our study during ventilation in supine position without capnoperitoneum. This may be due to the correlation between BMI and PEEP values needed to prevent alveolar collapse in supine position observed in our study. Nestler and colleagues suggested even higher PEEP levels for prevention of atelectasis formation based on intraoperative EIT measurements in obese patients undergoing laparascopic surgery in supine position [21].

Some intra-individual variation of Open Lung and Best Compromise PEEP was observed in supine as well as in Trendelenburg position. This suggests that intraoperative monitoring of regional ventilation with EIT on an individual basis could contribute to improvement of lung-protective ventilation strategies. This may not be cost-effective for patients with general low risk of PPC and operation in supine position, but might be of benefit in high risk patients and body positions with increased intra-abdominal and transpulmonary pressure.

### Strengths

Our study has some strengths. To our knowledge, it is the first study that assessed Open Lung PEEP and Best Compromise PEEP with EIT both after induction of general anesthesia and in Trendelenburg position with capnoperitoneum. Previous studies performing PEEP titration with EIT did so after induction of general anesthesia but not after establishment of capnoperitoneum [15, 21, 22]. This limits the generalizability of their findings to the markedly different respiratory mechanics in patients with capnoperitoneum. The method for identifying Open Lung PEEP and Best Compromise PEEP used in our study detects both overdistension and alveolar collapse, while some previously used EIT methods are measures for alveolar collapse but are relatively insensitive to overdistension [21, 22]. With this approach, we were able to show that the PEEP levels required to prevent alveolar collapse and to provide equal balance between collapse and overdistension are positively correlated to BMI in the supine position but not in the Trendelenburg position with capnoperitoneum. The latter is an intriguing new finding that merits further investigation. We derived regression equations for calculation of Open Lung PEEP and Best Compromise PEEP according to BMI in the supine position, enabling clinicians that have no EIT available to benefit from our results.

### Limitations

Our study has several limitations. As this study was conducted in the department of gynecology and obstetrics, only younger female patients were included, limiting the generalizability of our findings. Our findings should be examined in a mixed gender cohort, to eliminate possibility of gender specific findings. Moreover, as patients with BMI > 35 kg/m^2^ were excluded from the present study, our results cannot be extrapolated to morbidly obese patients.

In general, the perioperative risk of pulmonary complications is low in a relatively young and healthy patient population as studied in our manuscript. Therefore, it is unclear whether individualized adjustment of PEEP will have any clinically relevant effects on patient-centered outcomes in this patient population.

The effects of Trendelenburg position and capnoperitoneum were not investigated separately. The sample size was relatively small and was further reduced by a drop-out rate of 10%. However, given the relatively homogeneous patient population, we were still able to obtain robust values for median and interquartile ranges of Open Lung and Best Compromise PEEP. Post hoc power calculations using the actually achieved effect sizes revealed a statistical power of > 0.95 for detecting a difference in PEEP level between supine position and Trendelenburg position.

Our study was neither designed nor powered to investigate the influence of EIT-guided PEEP postoperative pulmonary complications. Nevertheless, none of the patients investigated in this study had additional oxygen requirements or needed invasive or non-invasive mechanical ventilation after the end of surgery.

A randomized study to assess the effect of EIT-guided PEEP settings during Trendelenburg position with capnoperitoneum on the occurrence of PPCs would be desirable to confirm our results and further specify the optimal PEEP level for prevention of PPCs after mechanical ventilation during general anesthesia.

## Conclusions

During mechanical ventilation in Trendelenburg position with capnoperitoneum, higher PEEP levels of 16 to 20 cmH_2_O do not lead to relevant pulmonary overdistension and could be considered for preventing regional pulmonary hypoventilation and alveolar collapse. In supine position without capnoperitoneum, significantly lower PEEP levels that could be adjusted according to BMI may be suitable for achieving a balanced compromise between alveolar collapse and overdistension.

## Data Availability

The datasets generated and analysed during the current study are not publicly available for legal reasons (European Union General Data Protection Regulation). However, anonymized data can be made available from the corresponding author on reasonable request.

## Abbreviations

BMI: Body Mass Index
C_rs_: Respiratory System Compliance
EIT: Electrical Impedance Tomography
MV: Mechanical Ventilation
PPC: Postoperative Pulmonary Complications
PEEP: Positive End-Expiratory Pressure
SpO_2_: Peripheral oxygen saturation by pulse oxymetry
